# Targeting under-diagnosis in hereditary hemorrhagic telangiectasia: a model approach for rare diseases?

**DOI:** 10.1186/s13023-014-0115-7

**Published:** 2014-07-25

**Authors:** Giuseppe A Latino, Dale Brown, Richard H Glazier, Jonathan T Weyman, Marie E Faughnan

**Affiliations:** 1 Toronto HHT Program, Department of Medicine, Division of Respirology, St. Michael’s Hospital, Toronto, Canada; 2 Li Ka Shing Knowledge Institute of St Michael’s Hospital, Toronto, Canada; 3 Toronto HHT Program, University of Toronto, Toronto, Canada; 4 Department of Pediatrics, The Hospital for Sick Children, 555 University Avenue, Toronto M5G 1X8, Ontario, Canada; 5 Department of Otolaryngology/Head & Neck Surgery, Princess Margaret Hospital, University Health Network, Toronto, Canada; 6 Department of Family and Community Medicine, St. Michael’s Hospital and University of Toronto, Toronto, Canada; 7 Institute for Clinical Evaluative Sciences, Toronto, Canada

**Keywords:** Hereditary hemorrhagic telangiectasia (HHT), Osler-Weber-Rendu, Under-diagnosis, Prevalence, Ears, Nose and throat (ENT), Rare disease, Epistaxis

## Abstract

**Background:**

Hereditary hemorrhagic telangiectasia (HHT), a rare autosomal dominant disease, is considered under-diagnosed. Our primary objective was to provide evidence of under-diagnosis of HHT in a North American population. We hypothesized that variation would exist in the diagnosed prevalence (D-prevalence) across regions in the province of Ontario, Canada and across age groups, due to under-diagnosis in certain groups. Our secondary objective was to collect data regarding contact and local access to consult specialists by HHT patients to help guide potential future diagnostic programs.

**Methods:**

*Primary objective-* 556 adult patients with a definite HHT diagnosis seen at the Toronto HHT Centre were identified and geocoded with postal codes. Prevalence rates were calculated using Canadian census data. *Secondary objective*- A driving network model was developed in ArcGIS. Service area buffers around ear, nose and throat (ENT) clinics in Ontario were generated to evaluate the proportion of the Ontario population with access to these clinics. A survey was also sent to the email contact list of HHT Foundation International, targeting people with diagnosed HHT, regarding consultation with ENT physicians for epistaxis and timing of HHT diagnosis.

**Results:**

*Primary objective*- D-prevalence rates varied among regions, from no cases to 1.1 cases per 5000 in large Ontario cities. There were no significant differences between urban and rural prevalence rates. Variation in prevalence was seen across age groups, with greater prevalence in older adults (≥50 years-old) compared with adults 20–49 years-old (0.36 versus 0.26 per 5000, p < 0.0005). *Secondary objective-* Most Ontarians had access to ENT clinics within a 30, 60 and 90 minute modeled drive time (92.7%, 97.8% and 98.6%, respectively). Nearly 40% of surveyed patients consulted an ENT physician for their epistaxis, on average 13.9 ± 12.2 years prior to being diagnosed with HHT.

**Conclusions:**

The prevalence of HHT in Ontario is highly variable across regions and age-groups, suggesting under-diagnosis. Given that patients with HHT frequently consult ENT physicians for epistaxis prior to HHT diagnosis, and that there is almost universal access to ENTs in Ontario, we propose targeting ENT clinics as a province-wide approach to detect undiagnosed HHT patients and families.

## Background

**Figure 1:**
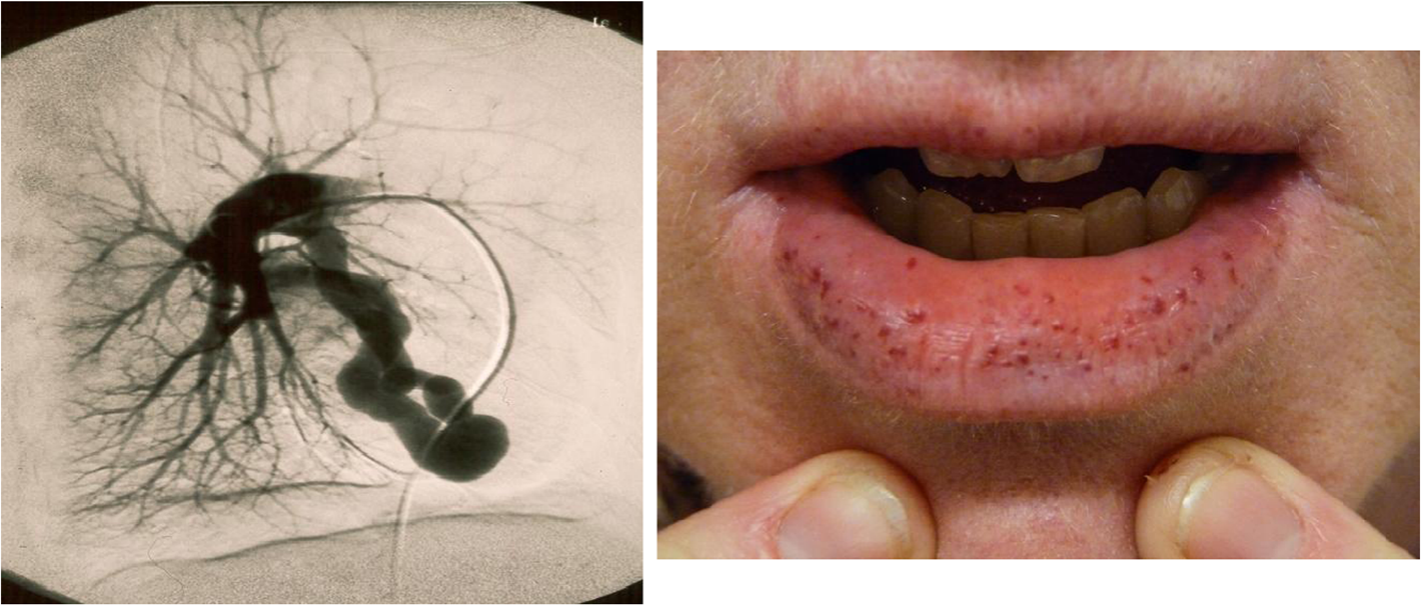
**Clinical manifestations of HHT, including pulmonary arteriovenous malformations (left) and mucocutaneous telangiectases (right).** According to the Curaçao criteria, a confirmed clinical diagnosis of HHT requires 3 or more of epistaxis, mucocutaneous telangiectasia, visceral AVMs, and a positive family history of HHT. Recurrent epistaxis is the most common symptom of HHT and is present in more than 90 % of adults ≥50 years of age. HHT may also be diagnosed genetically based on known disease-causing mutations.

Hereditary hemorrhagic telangiectasia (HHT) is an autosomal dominant disorder of vascular dysplasia. It is characterized by organ arteriovenous malformations (AVMs) of mainly the lungs, central nervous system and liver, as well as telangiectases of the dermis and mucous membranes (Figure 1) [[1]–[3]]. Both AVMs and telangiectases are prone to bleeding leading to chronic epistaxis, the most common symptom of HHT, as well as chronic gastrointestinal bleeding and life-threatening pulmonary or cerebral hemorrhage in some patients. AVMs and telangiectases consist of direct artery-to-vein connections, and therefore also put patients at risk of life-threatening complications due to shunting, such as stroke, brain abscess and high-output cardiac failure [[1],[3]–[7]].

The diagnosis of HHT in a family is based first on the clinical diagnosis of HHT of an index case using the Curaçao Criteria. Once the index case is diagnosed, the next step is to identify the disease causing HHT mutation in that patient. First-degree relatives can be diagnosed using clinical criteria if symptomatic, or by testing for the familial mutation if asymptomatic or paucisymptomatic [[1],[3]]. The two common genes implicated in HHT are the endoglin gene (*ENG* or HHT1) and the activin A receptor type II-like 1 gene (*ALK-1* or HHT2), both encoding endothelial cell surface proteins of the TGF-β/BMP-9 signaling pathway [[8]–[10]]. A third gene, *SMAD4,* has been recently discovered in a small subset of patients with HHT, primarily in those with an overlap syndrome with juvenile polyposis [[11]]. Overall, a disease-causing mutation can be identified in approximately 80% of HHT patients, but almost every family has a private mutation [[12],[13]].

HHT has a broad geographic distribution and is found widely in many ethnic and racial groups, with an estimated prevalence of at least 1 in 10,000 [[1],[2],[14]–[19]]. To date, there have been no North American epidemiologic studies reporting HHT prevalence outside of an estimate by our group using administrative billing data [[20]]. As is the case for many rare diseases, expert opinion is that HHT is under-diagnosed, though this has not been studied in North America. This expert opinion, however, is based on the ongoing HHT Centre experience of regularly identifying new adults and families with HHT. There is also preliminary evidence in the literature suggesting that HHT is indeed under-diagnosed in Europe [[21]], and that diagnosis is often delayed and made only after serious complications of the disease arise [[22]].

Given the hereditary nature of HHT, as well as the very high penetrance of the disease, there should theoretically be little-to-no variability in the *true* prevalence of the disease across various age groups. However, given its likely under-recognition and age-related expression, we expect there to be variability in the actual *diagnosed* prevalence of HHT, as suggested in a recently reported study of HHT diagnoses [[20]]. Thus, our primary objective was to provide evidence of under-diagnosis of HHT in North America by estimating the prevalence of the disease in Ontario, Canada using our provincial HHT Centre database and population statistics. We hypothesized that there would be variation in the diagnosed prevalence (D-prevalence) across regions in the province and across age groups due to under-diagnosis in certain populations. By demonstrating this, we could then begin to study approaches to resolving under-recognition of HHT in North America.

Our secondary objective was to collect data regarding specialist consultation amongst HHT patients, and model access to appropriate specialists in order to help guide potential future diagnostic programs. Given that recurrent epistaxis is almost universal among adults with HHT, we surveyed HHT Foundation International contacts about their consultation with emergency room (ER) and ear, nose and throat (ENT) physicians, for epistaxis, prior to their diagnosis of HHT. In addition, we estimated geographic access to ENT physicians across Ontario by developing a driving network model of the Ontario population’s access to ENT physicians.

It is our hope that this approach of detecting under-diagnosis and developing targeted strategies to improve diagnostic rates may serve as a potential model for other rare diseases.

## Methods

### D-prevalence of HHT in Ontario, Canada

The Toronto HHT Centre is a specialized HHT Centre of Excellence (the only one in Ontario, Canada) that provides expert multidisciplinary care to HHT patients and families with suspected or confirmed HHT. The Toronto HHT Database is a FileMaker Pro relational database, which includes all of those patients assessed at the Toronto HHT Centre from 1997 onwards. A comprehensive history and physical examination was completed by an HHT specialist for all patients, as well as routine screening for pulmonary and cerebral AVMs. Genetic testing was recommended to all families, which most did pursue, in order to identify a potential familial HHT mutation. Consent was obtained from all patients to include their clinical information in the Toronto HHT database.

All adult patients with a definite clinical or genetic diagnosis of HHT from the Toronto HHT Centre were identified (N = 627). Postal codes were then recorded for all but 36 patients (35 deceased, 1 missing address). The remaining 591 patients were geocoded using their postal codes, with 35 being excluded (28 patients lived outside of Ontario, 7 patients could not be geocoded). In total, 556 (98.5%) patients living in Ontario were successfully geocoded. The population of Ontario was determined using 2006 Canadian census data and was used to calculate the D-prevalence of HHT in the province.

In order to identify any potential causes for under-diagnosis, the D-prevalence of HHT in urban settings was compared to that of rural areas in Ontario. All census subdivisions (CSDs) with a population of ≥1000 within Ontario Census Metropolitan Areas (CMAs), as defined by Statistics Canada, were identified (n = 125) and used to represent the urban study area. The remaining CSDs in Ontario represented the rural study area. For each urban CSD and for the rural portion of Ontario, HHT patient counts were calculated for 2 age groups (20–49 and ≥50 years-old) and the D-prevalence of HHT within each age group was then calculated using aged-match total population counts from the 2006 Census of Canada. The ≥20 years-old cut-off was used since the Canadian Census reports age-related population data by decade only, and did not include isolated population data on adults less than 20 years old. The ≥50 years-old cut-off was chosen since previous studies have shown that at least 90% of patients with HHT are symptomatic by this age [[4]].

In order to eliminate overestimation biases due to large families living in small cities, D-prevalence rates were also compared specifically between large Ontario cities, defined as those cities with a population ≥50,000, and very large cities, defined as cities with a population ≥250,000.

### Specialist consultation by patients with HHT

A web-based survey targeting physician-diagnosed HHT patients was distributed to all HHT Foundation International email contacts (N = 2836). Demographic information including sex, present age, and age at time of HHT diagnosis were obtained. Participants with a self-reported but physician-determined diagnosis of HHT were asked about the presence of recurrent epistaxis. In those reporting epistaxis, further survey questions enquired about specialist consultation with ENT and ER physicians prior to their HHT diagnosis.

### Access to ENT clinics across Ontario

A driving network model for all roads in Ontario was developed in ArcGIS, a geographic information system used to create maps, and compile and analyze geographic data. An estimated drive time for each road segment was estimated using non-rush hour speed limits, road length, and elevation change. Direction of travel restrictions were implemented for one-way streets and divided highways. The model also included impedances for travel delay at intersections, ranging from 5 to 30 seconds depending on the direction of travel (i.e. left or right turn, proceed straight) and the type of road (i.e. primary, secondary, local).

Using postal codes retrieved from the Ontario Medical Association as well as the College of Physicians and Surgeons of Ontario websites, locations of ENT clinics were geocoded. Service area buffers were then generated around each clinic to model the area which could be traveled to within a 30, 60 and 90 minute drive in any direction from the clinic location. Service area buffers were then combined for each time category to create three large buffers of the entire area in Ontario in which residents could access an ENT clinic within a 30, 60 or 90 minute drive.

Identifiers were then assigned to 2006 census dissemination areas (DAs) based on whether their geometric center point (i.e. centroid) was located within the buffer for a given time. Total populations were assigned to each DA based on the 2006 Census of Canada. The total number of Ontarians within a 30, 60 and 90 minute drive of an ENT clinic was then determined.

### Statistical analysis

GraphPad Prism, a commercial scientific 2D graphing and statistical software, was used to generate all graphs and statistical analysis. Chi-square test was used to calculate statically significant differences in D-prevalence rates of HHT across cities, in urban versus rural centers, as well as differences in D-prevalence between various age groups across Ontario.

### Ethics

Ethics approval for this study was obtained from the St. Michael’s Hospital Research Ethics Board in Toronto, Ontario, Canada. Previous informed consent was given by all patients included in our Toronto HHT Centre database.

## Results

### Variability of D-prevalence across all cities in Ontario

**Figure 2:**
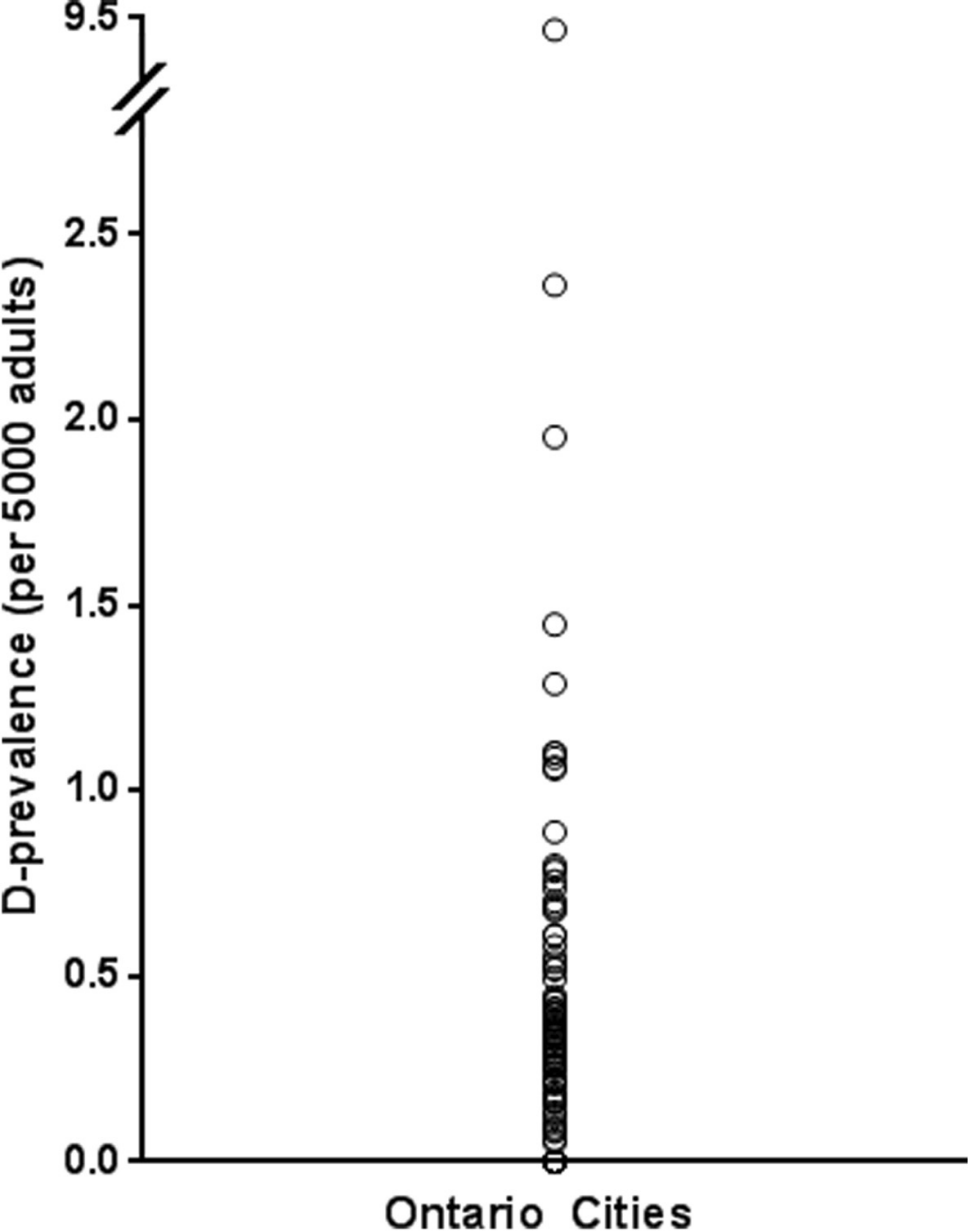
**Variation in D-prevalence across all cities in Ontario, Canada.** Each circle represents a given D-prevalence from one or more Ontario cities. There were 59 cities with no reported cases of HHT. The D-prevalence of HHT varied significantly across included regions and ranged from no cases to 9.4 per 5000 adults.

In total, there were 556 adult (≥20 years old) patients in the Toronto HHT Database with a confirmed diagnosis of HHT and living in the province of Ontario. There were 9,116,380 adults (≥20 years) living in Ontario in 2006, making the D-prevalence of HHT approximately 0.31 per 5000 people. The D-prevalence varied significantly across all regions of Ontario from no cases in 59 cities to as high as 9.4 cases per 5000 people in one smaller city (Figure 2). There were no significant differences between overall urban and rural D-prevalence rates (0.31 versus 0.30 per 5000, p = 0.79).

### Variability of D-prevalence in large cities in Ontario

**Figure 3:**
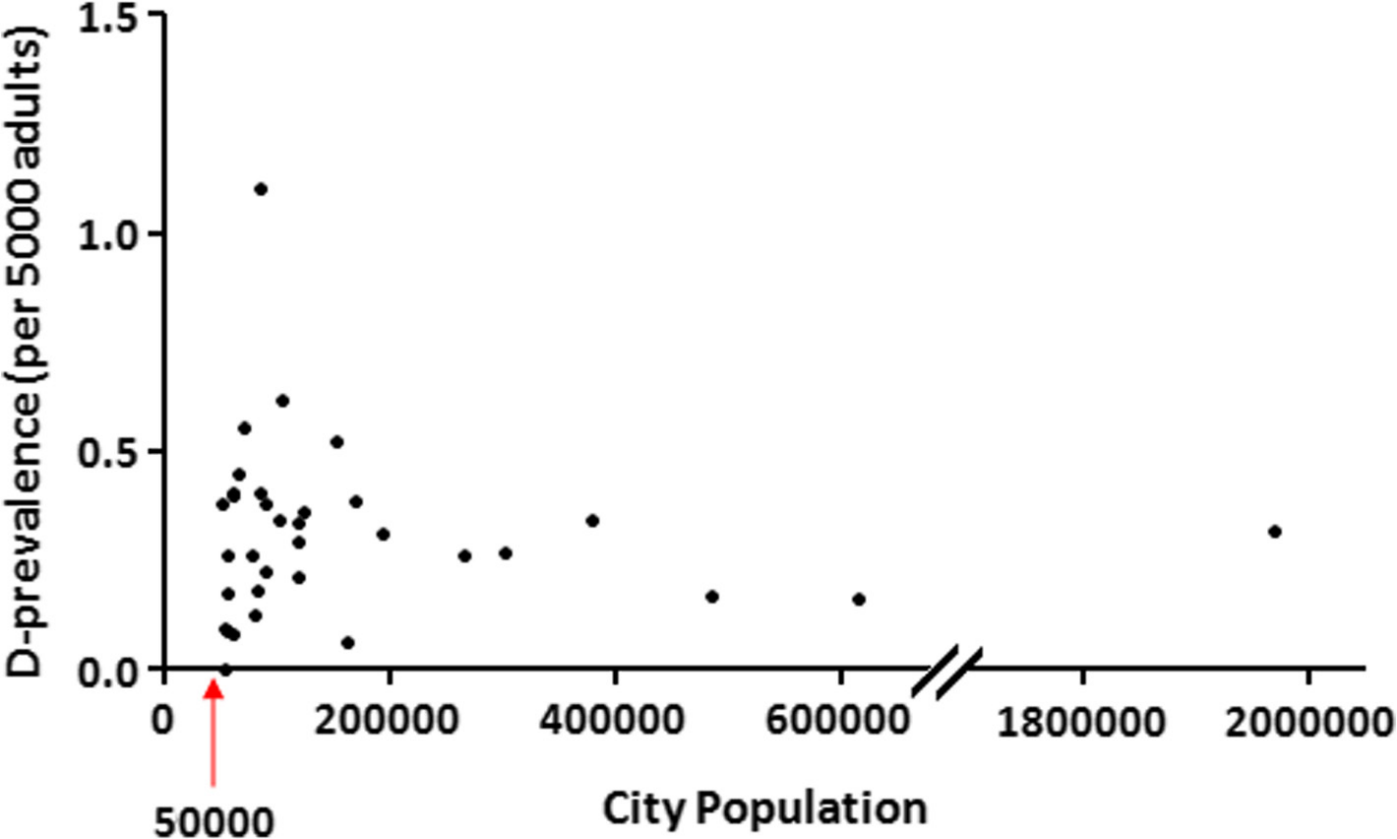
**City size versus D-prevalence in large cities in Ontario, Canada.** In order to eliminate overestimation biases due to large families with HHT living in small cities, the D-prevalence of HHT was compared in large cities, defined as those centers with a population ≥50,000. Even amongst these cities, there was still significant variation in the D-prevalence across regions, which ranged from no cases to 1.1 cases per 5000 adults. There was no correlation between the population size and the D-prevalence (R^2^ = 0.0005).

Of the 34 large cities (population ≥50,000 adults) in Ontario, there was no correlation (R^2^ = 0.0005) between the population size and the D-prevalence, which ranged from no cases to 1.1 cases per 5000 adults (Figure 3).

### Variability in D-prevalence across age groups

**Figure 4:**
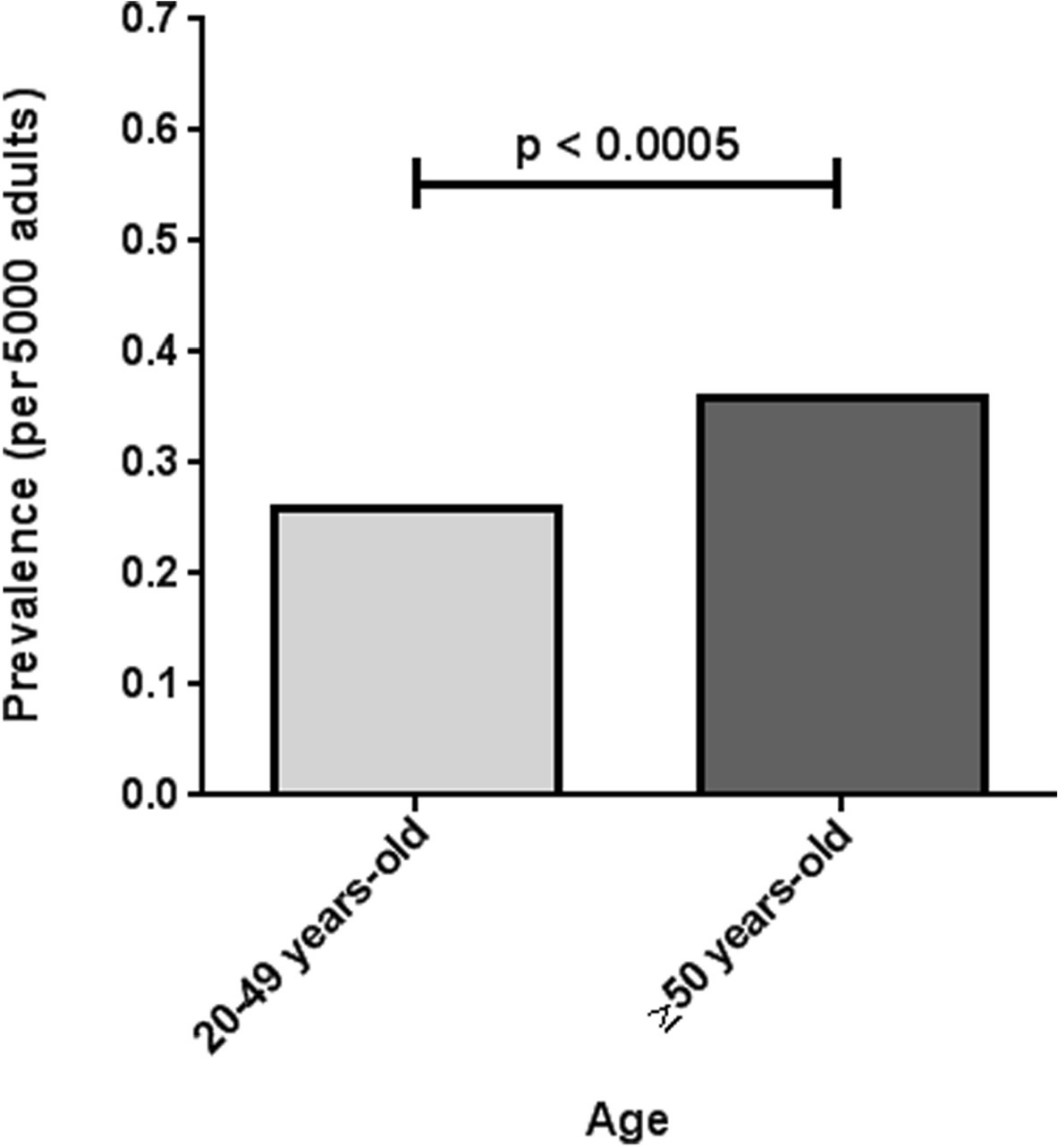
**Variation in D-prevalence across age-groups in all cities in Ontario, Canada.** D-prevalence rates were significantly higher in older adults (0.36 ± 0.32 per 5000) compared to younger adults (0.26 ± 0.22 per 5000).

Of the 556 adults with confirmed HHT, 275 were 20-49 years-old and 281 were ≥50 years-old. D-prevalence rates were significantly higher in older adults compared to younger adults (0.36 versus 0.26 per 5000, respectively, p < 0.0005) (Figure 4). This difference held true even for the very large Ontario cities (population ≥250,000 adults), where the overall D-prevalence was 0.28 per 5000. In these cities, the D-prevalence was 0.22 versus 0.38 per 5000 for the 20–49 and ≥50 years-old age groups, respectively (p < 0.00005) (Table 1). Very similar results were found for large Ontario cities (population ≥50,000 adults).

**Table 1:** Variation in D-prevalence across age groups in very large cities in Ontario, Canada

**City**	**Population size**	**Total number of confirmed HHT cases**	**D-prevalence for 20–49 year-olds (per 5000)**	**D-prevalence for ≥50 year-olds (per 5000)**
London	267,145	14	0.22	0.31
Brampton	303,700	16	0.20	0.40
Hamilton	379,780	26	0.24	0.48
Mississauga	486,270	16	0.16	0.17
Ottawa	614,770	20	0.16	0.16
Toronto	1,947,455	134	0.25	0.48
**Total**	**3,999,120**	**226**	0.22*	0.38*

Note: the combined adult population of these cities comprises 50 % of the entire adult population of Ontario; *denotes a statistically significant difference of p < 0.00005.

### Survey of HHT foundation international HHT patients

940 participants with a self-reported physician-confirmed diagnosis of HHT completed the web-based survey, 79% of whom were North American. A large majority of participants (86%) reported a history of recurrent epistaxis and nearly 40% had consulted an ENT physician for epistaxis prior to being diagnosed with HHT. The mean reported time between this initial consult and HHT diagnosis was 13.9 ± 12.2 years (Table 2).

**Table 2:** Survey of HHT patients regarding consultation for epistaxis with ENT specialists and ER physicians and relative timing of HHT diagnosis

Proportion of respondents (%)	940/2836 (33 %)
Mean age ± SD at time of survey	54.1 ± 14.1 years
Mean age ± SD at time of diagnosis	37.1 ± 15.5 years
Proportion of females (%)	630/940 (67 %)
Proportion of North Americans	738/940 (79 %)
Proportion of patients with recurrent epistaxis (%)	807/940 (86 %)
Proportion of patients with ENT consult prior to HHT diagnosis (%)	349/940 (37 %)
Proportion of patients with ER consult prior to HHT diagnosis (%)	154/940 (16 %)
Mean age ± SD at first ENT consult	27.8 ± 16.8 years
Mean age ± SD at first ER consult	30.1 ± 17.4 years
Mean interval of time ± SD from initial ENT consult to HHT diagnosis	13.9 ± 12.2 years
Mean interval of time ± SD from initial ER consult to HHT diagnosis	11.3 ± 12.6 years

### Access to ENT clinics in Ontario

**Figure 5:**
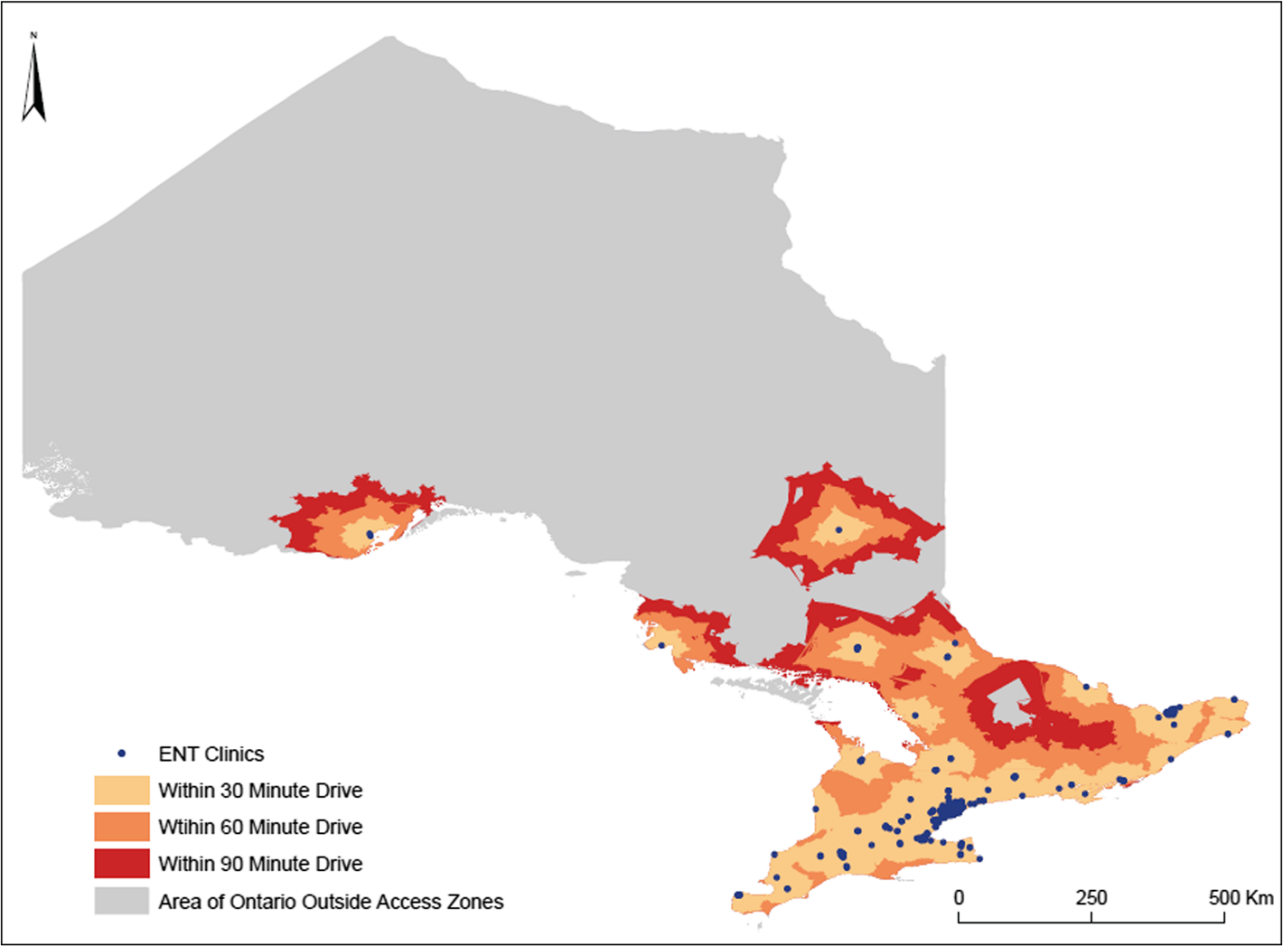
**Access to ENT clinics in Ontario, Canada.** The vast majority of adult Ontarians have access to an ENT clinic within a 30, 60 and 90 minute modeled drive time (92.7 %, 97.8 % and 98.6 %, respectively. While the majority of Northern Ontario appears outside the access zones (grey), only a very small proportion (<10 %) of the population inhabits this region of the province.

The vast majority of the adult Ontario population had access to an ENT clinic within a 30, 60 and 90 minute drive (92.7%, 97.8% and 98.6%, respectively) (Figure 5).

## Discussion

In this study, we have provided data supporting the under-diagnosis of HHT in Ontario. We have also shown almost universal access to ENT clinics for the Ontario population and evidence that HHT patients are consulting ENT physicians for epistaxis, both of which support an approach targeting ENT physicians in improving the diagnostic rates of HHT. For many patients, there is a substantial delay between their initial ENT consult and receiving a diagnosis of HHT. This lapse represents a significant opportunity to intervene and reduce both the morbidity and mortality associated with HHT.

We have demonstrated a D-prevalence of HHT in Ontario of approximately 0.31 per 5000 individuals. However, there is also good evidence supporting under-diagnosis in the province. The D-prevalence of HHT varied widely between cities in Ontario, and could not be explained by differences in urban versus rural areas. In order to eliminate overestimation biases due to large families living in small cities, D-prevalence rates were compared specifically between cities with a population ≥50,000. Even in these larger centers, many of which had tertiary hospitals and specialized expertise, there was still significant variation in the D-prevalence across cities.

Moreover, the D-prevalence of HHT was significantly greater in older adults compared with younger ones in the province. Given the genetic nature of the disease, all patients with HHT are born with the disorder and genetic testing is available for pre-symptomatic diagnosis. As such, a difference in D-prevalence between age groups should not be seen unless HHT is being under-recognized in some age groups. From a clinical perspective, many of the symptoms of HHT, including recurrent epistaxis and telangiectasia, become more prevalent with age and are most consistently present by age 50 years, making the clinical diagnosis of HHT easier. In contrast, genetic testing is often necessary for confirmation of the diagnosis in younger adults and children, many of whom may be asymptomatic or have only mild symptomatology but are still at risk for AVM complications [[1],[4],[5],[23]–[27]]. This may partially explain the difference in D-prevalence between the different age groups and the under-diagnosis of HHT in younger adults.

Using the current estimated D-prevalence of HHT in Ontario (0.31 per 5000) and the highest D-prevalence seen in a large Ontario city (1.1 per 5000) to approximate the true province-wide prevalence, it is estimated that as many as 70% of the HHT cases are undiagnosed in Ontario. This represents an estimated 1400 individuals in Ontario who are unaware of their diagnosis and are at risk of life-threatening, but preventable, complications.

Given the nearly universal access to ENT clinics for Ontarians, we believe that ENT physicians are an excellent target to improve the diagnostic rates of HHT. Recurrent epistaxis is the most common symptom of HHT, occurring in approximately 90% of patients over 50 years-old [[1],[4],[5]]. Recurrent epistaxis is, therefore, a very sensitive clinical biomarker of HHT. Individuals with HHT often seek the care of ENT specialists or ER physicians in managing their epistaxis. In our web-based survey, nearly 40% of participants consulted an ENT physician, most as young adults, prior to being diagnosed with HHT. However, the time between this initial consult and receiving a diagnosis was a staggering 14 years. This represents a critical period in which serious complications of HHT can occur, and a time in which individuals may choose to have children who are at a 50% risk of inheriting the disease. While recurrent epistaxis may not be a very specific clinical biomarker, since it is a symptom shared by many diseases, it is highly sensitive and would capture the majority of patients with undiagnosed HHT. This may prove useful in any campaign focused on improving diagnostic rates and reducing disease-related mortality and morbidity. To accomplish this, further work, in conjunction with ENT specialists, will be required to determine effective, efficient and feasible methods of delineating HHT-related epistaxis from non-HHT-related epistaxis.

It is important to acknowledge the limitations of this study. As the only HHT Centre for adults in Ontario, we assumed that the Toronto HHT Centre manages the majority of patients with known HHT in the province. However, there may be variable referral rates to our institution, with variation in D-prevalence across cities being somewhat due to referral patterns. For instance, some academic centres outside of Toronto may be undertaking management of patients with the disease, which may partly explain the lower D-prevalence rates from certain major Ontario cities like Ottawa. However, these postulated differences in referral patterns are likely to impact patient capture from all ages and cannot explain the variation in D-prevalence seen amongst different age groups, suggesting that under-diagnosis is a true concern. Moreover, it is plausible that differences seen in D-prevalence between Ontario cities may be partly accounted for by settlement patterns and the Founder effect, as reported in previous HHT studies [[28],[29]]. We accounted for this by comparing the D-prevalence of HHT between large cities in an attempt to reduce overestimation bias both when calculating D-prevalence and estimating the percentage of undiagnosed HHT cases. The variation in D-prevalence seen across large cities and across age groups cannot be accounted for by settlement patterns alone and is once again suggestive of under-diagnosis. However, it is important to acknowledge that while upwards of 70% of HHT cases may be undiagnosed in Ontario, this estimate captures both unidentified HHT cases as well as patients with suspected disease in which a definite diagnosis has been difficult to make. Although this may account for some of the differences in D-prevalence, especially between age groups, it is likely to be of minimal impact given that the majority of our patients with suspected HHT are negative for a known disease-causing mutation. Next, the web-based survey used is limited by both response bias, since individuals without access to a computer or email were excluded, and recall bias. Driving times were calculated under minimal to no traffic conditions, which is an unlikely occurrence. Regardless, even with the addition of a 30 to 60 minute traffic delay, nearly 93% of Ontarians are within a 60 to 90 minute drive of an ENT clinic. Finally, access to ENT clinics was calculated under the assumption that all Ontarians drive or own a motor vehicle, and the reported access rates are thus likely to be a slight overestimate.

The *Rare Diseases Act of 2002* defines a rare disease as one that affects less than 200,000 people in the United States, with more than 6000 diseases meeting this criterion [[30]]. According to the National Organization for Rare Disorders (NORD), approximately 1 in 10 Americans have a rare disease, an astounding rate that is comparable to major illnesses like diabetes mellitus. Patients with rare diseases often receive multiple and incorrect diagnoses for years before ever receiving the correct one [[14],[22],[31],[32]]. In this time, serious complications associated with the disease may occur, which is our concern with respect to HHT. By implementing strategies to improve the recognition and diagnoses of HHT, we may be able to intervene and help guide patients to the resources and specialized management that they require. This may also lead to subsequent diagnoses in several other family members, especially in autosomal dominant disorders like HHT. We speculate that these same approaches might be relevant for other hereditary rare diseases as well.

In this article, we have presented a novel approach that we hope will help to improve the diagnostic rates of HHT and serve as an example for other rare diseases. This innovative model consists of: 1) providing evidence of under-diagnosis; 2) identifying a sensitive clinical biomarker (e.g. recurrent epistaxis) in the disease; 3) targeting specialists that are consulted for this biomarker; 4) ensuring patients have access to these specialists; and 5) developing disease awareness programs and diagnostic initiative strategies to improve the recognition and diagnoses of a given rare disease. By detecting index cases, both symptomatic and asymptomatic family members may then be screened through clinical and genetic evaluation. Those affected by the disease may undergo subsequent screening and potentially preventative therapies, when available.

## Conclusion

By identifying variation in the D-prevalence of HHT across different cities and age groups in Ontario, we have provided evidence of the under-diagnosis of HHT in North America. Given that recurrent epistaxis is very common in HHT, it is a sensitive clinical biomarker in the disease. By exploiting this and collecting data regarding specialist consultation amongst HHT patients and population access to these specialists, we have identified ENT physicians as an excellent target group to involve for improving the diagnostic rates of HHT. The implication is that a province-wide detection program involving ENT physicians is feasible, with the goal of detecting index cases of HHT and unrecognized HHT families. While this study focused specifically on HHT, we believe that the strategies used here can be more generally employed as a model approach for identifying and improving under-diagnosis in other rare diseases.

## Abbreviations

AVM: Arteriovenous malformation

CSD: Census subdivision

CMA: Census metropolitan area

DA: Dissemination areas

D-prevalence: Diagnosed prevalence

ENT: Ears, nose and throat

ER: Emergency room

HHT: Hereditary hemorrhagic telangiectasia

NORD: National organization for rare disorders

## Competing interests

The authors declare that they have no competing interests.

## Authors’ contributions

GAL made substantial contributions to the design of the work, to the acquisition, analysis and interpretation of the data, and to the drafting and critical revising of the manuscript. DB made important contributions to the design of the work and critically revising the important intellectual content. RG made substantial contributions to the acquisition and analysis of data and provided critical input during the revision of the manuscript. JTW was critical to the design, acquisition, analysis and interpretation of the data, as well as provided substantial feedback for revisions of the important intellectual content. MEF provided key contributions to the conception and design of the work, the interpretation of data, and the drafting and revising of the manuscript. All authors read and approved the final manuscript.
